# Genome-Wide Analysis of Transposon and Retroviral Insertions Reveals Preferential Integrations in Regions of DNA Flexibility

**DOI:** 10.1534/g3.115.026849

**Published:** 2016-01-26

**Authors:** Pavle Vrljicak, Shijie Tao, Gaurav K. Varshney, Helen Ngoc Bao Quach, Adita Joshi, Matthew C. LaFave, Shawn M. Burgess, Karuna Sampath

**Affiliations:** * Division of Biomedical Sciences, Warwick Medical School, University of Warwick, Coventry CV4 7AJ, United Kingdom; † Temasek Life Sciences Laboratory, National University of Singapore, 117604, Singapore; ‡ National Human Genome Research Institute, Bethesda, Maryland 20892-8004

**Keywords:** transposon, Ac/Ds, genome-wide analysis, integrations, gene targeting, genome engineering, functional genomics, Tol2, retrovirus, MMLV, mouse, ES cells, vertebrate genomes, zebrafish

## Abstract

DNA transposons and retroviruses are important transgenic tools for genome engineering. An important consideration affecting the choice of transgenic vector is their insertion site preferences. Previous large-scale analyses of Ds transposon integration sites in plants were done on the basis of reporter gene expression or germ-line transmission, making it difficult to discern vertebrate integration preferences. Here, we compare over 1300 Ds transposon integration sites in zebrafish with Tol2 transposon and retroviral integration sites. Genome-wide analysis shows that Ds integration sites in the presence or absence of marker selection are remarkably similar and distributed throughout the genome. No strict motif was found, but a preference for structural features in the target DNA associated with DNA flexibility (Twist, Tilt, Rise, Roll, Shift, and Slide) was observed. Remarkably, this feature is also found in transposon and retroviral integrations in maize and mouse cells. Our findings show that structural features influence the integration of heterologous DNA in genomes, and have implications for targeted genome engineering.

DNA elements capable of genomic integration, such as transposons and retroviruses, are important tools in molecular biology research. From the fission yeast *Schizosaccharomyces pombe* to humans, these vectors have been used for gene delivery and insertional mutagenesis (*e.g.*, Cavazzana-Calvo *et al.* 2000; Aiuti *et al.* 2002; Kawakami and Noda 2004; Wang *et al.* 2007a; Guo *et al.* 2013). Significantly, the integration of these elements has revealed features of genes and genomes, such as the function and regulation of genes, and the “open” state of chromatin (Wang *et al.* 2007a; Genovesi *et al.* 2013; Guo *et al.* 2013; De Ravin *et al.* 2014; Davie *et al.* 2015; Rad *et al.* 2015; Takeda *et al.* 2015).

Three of the integrating elements currently used in zebrafish are the Tol2 and Activator/Dissociator (Ac/Ds) transposons, and the Moloney Murine Leukemia Virus (MMLV) (Lin *et al.* 1994; Koga *et al.* 1996; Kawakami *et al.* 2004; Emelyanov *et al.* 2006; McGrail *et al.* 2011; Song *et al.* 2012; Cheng *et al.* 2014; Quach *et al.* 2015). Ac/Ds and Tol2 transposable elements are members of the hAT family (named for hobo, Ac and Tam3) (Calvi *et al.* 1991). They integrate into the host DNA through a “cut-and-paste” mechanism requiring *cis*-terminal elements flanking the transgene of interest and the transposase enzyme, which may be encoded in the autonomous elements (*e.g.*, Ac) or exogenously supplied (*e.g.*, Ds) (McClintock 1951). Some of the features that make hAT transposons particularly amenable for transgenic work in zebrafish are the accurate mechanism of integration (with well-defined integration sequences), the ability to be remobilized if desired, the small size of *cis*-required sequences (∼600 bp), a reasonably high transposition frequency, and moderate copy numbers (Emelyanov *et al.* 2006). Significantly, transposable element vectors have a relatively large insert capacity (>10 kb) and are easy to generate in a standard molecular genetics laboratory.

In contrast to transposable elements, retroviral vectors such as MMLV have a limited insert packaging size (usually <8 kb) and their production requires specialized technical expertise. However, retroviruses are currently the most efficient way to make a large number of insertions in the zebrafish genome, producing a high number of integrations for a given experiment (Amsterdam *et al.* 2011). The high copy number potential and high mutation rate were key features in the successful use of the MMLV retroviral vector in an insertional mutagenesis screen that targeted a large number of protein coding genes in the zebrafish genome (Varshney *et al.* 2013).

One important consideration affecting the choice of transgenic vector is their insertion site preferences. Integrations occurring in the 5′-end of genes are advantageous for creating insertional mutants. However, vectors that only target actively transcribed gene regions have limited use in capturing genes that are expressed at low levels, or those regulated by alternative promoters and enhancers. Similarly, while targeting enhancer regions might be an advantage for enhancer traps and detecting open chromatin, transgene expression might suffer from the variability imposed by positional effects (Roberts *et al.* 2014). Moreover, targeting of specific repetitive elements might lead to transgene inactivation, while targeting of 3′-UTR sequences might lead to changes in posttranscriptional regulation (Goll *et al.* 2009; McGaughey *et al.* 2014; Shpiz *et al.* 2014).

Integration bias has been reported for transposons and retroviruses in a number of systems (*e.g.*, Vigdal *et al.* 2002; Wu *et al.* 2003, 2005; Faschinger *et al.* 2008; Linheiro and Bergman 2008; Liang *et al.* 2009; Vollbrecht *et al.* 2010). Integration target sites are thought to be relatively random at large genomic scales, although there have been reports of association with genetic elements such as transcriptional start sites, strong enhancers or promoters, UTRs, and CpG islands (Wu *et al.* 2003; Kondrychyn *et al.* 2009; Vollbrecht *et al.* 2010; LaFave *et al.* 2014). At the nucleotide sequence level, different integrating elements have shown various degrees of sequence bias. MMLV shows a weak preference for T/A nucleotides just outside the 4 bp site of integration (LaFave *et al.* 2014). In contrast, Ac/Ds and Tol2 are not reported to show specific integration motifs (Kawakami 2007; Kondrychyn *et al.* 2009; Vollbrecht *et al.* 2010). However, these analyses have been limited by the number of genomic features analyzed, and the reliance on a small number of sites in some studies. Crucially, the available insert collections generated in zebrafish were either selected based on reporter gene expression or phenotypes, and only represent integrations that have been incorporated in the germ-line (Kondrychyn *et al.* 2009; Kawakami *et al.* 2010; LaFave *et al.* 2014).

We recently described the use of an Ac/Ds transposon system for a mutagenesis screen in zebrafish (Quach *et al.* 2015). We produced a collection of 642 transgenic lines marking distinct cell and tissue types, and mutagenized genes in the zebrafish genome by trapping and prematurely terminating endogenous protein coding sequences. Significantly, our gene/enhancer trap mutagenesis screen provides an unprecedented amount of Ds integration data in zebrafish.

In this study, we set out to analyze the genome-wide integration preferences of Ds in zebrafish. We examined how Ds integrations compare to those of other popular tools for generating transgenic zebrafish (Tol2 transposon and MMLV retrovirus), from the chromosome to the sequence level, and generated an unselected Ds integration set to explore the effect of selection on integration site preferences. We find that Ds integrations are more broadly distributed across gene regions than Tol2 and MMLV, and reporter-based selection does not affect integration site characteristics. Interestingly, while a strict motif at the target site was not found, we observed a preference for structural features correlated with DNA strand flexibility in the target DNA, which we also found in Ds integrations in maize, and in transposon and retroviral integrations in mouse ES cells.

## Materials and Methods

### Ds integration lines

The Ds integration lines were generated as described previously (Quach *et al.* 2015). Briefly, we coinjected one-cell stage zebrafish embryos with pDsDELGT4 (a gene and enhancer trap construct flanked by Ds sequences) together with *in vitro* transcribed Ac transposase mRNA. The resulting founder lines were then selected based on expression of either EGFP or mCherry indicating successful enhancer and gene trapping, respectively. To study Ds integration preferences in the absence of external selection, pDsDELGT4 plasmid was microinjected into 576 embryos at the one-cell stage as described above. Injected embryos were grown for 3–5 d to obtain sufficient DNA, pooled into groups of 6 and subjected to next-generation sequencing (NGS).

### Identification of Ds integration sites by TAIL-PCR and genomic sequencing

In order to identify unique Ds insertion sites, we first used thermal asymmetric interlaced PCR (TAIL-PCR) on expression-selected lines as previously described (Quach *et al.* 2015). Flanking sequences obtained from TAIL-PCR were analyzed against the zebrafish reference genome (Zv9) using BLAT (Kent 2002). Flanking sequences were considered unambiguously mapped if the entire TAIL-PCR generated sequence matched a single location of the genome assembly with 85% identity or more. This identity cut-off was derived empirically to account for differences between the AB strain used for Ds insertions and the Tubingen reference genome, and sequence quality derived from the TAIL-PCR protocol. Any ambiguously mapping TAIL-PCR-derived sequence was excluded from further analysis.

TAIL-PCR results represented only ∼75% of sites expected by Southern blot analysis. To determine insertion sites for the lines that could not be resolved by TAIL-PCR, NGS was performed on the Illumina MiSeq as described (Varshney *et al.* 2013) with the following modifications. About 500 ng of genomic DNA was fragmented using three pairs of restriction enzymes (*Mse*I/*Pst*I, *Bfa*I/*Ban*II, and *Csp*6I/*Eco*24I) in parallel. The digested samples were pooled and ligated with DNA linkers, and amplified by linker-mediated PCR using linker and Ds specific primers to capture the adjacent genomic DNA sequences. The Ds/gDNA/linker amplicons were subsequently ligated to Illumina paired-end adapters and sequenced. The first round of PCR was performed using a 3′-Ds ITR primer and a linker primer (5′-TATGAAAATGAAAACGGTAGAGGTATTTTACCGACCG-3′ and 5′-GTAATACGACTCACTATAGGGCACGCGTG-3′, respectively) and the second round of PCR was performed using nested 3′-Ds ITR and linker primers (5′-TTTACCGACCGTTACCGACCGTTTTCATC-3′ and 5′-GCGTGGTCGACTGCGCAT-3′, respectively). Ds insertion sites were identified using a modified version of the GeIST program previously used to detect MMLV LTR sequences (LaFave *et al.* 2014). For NGS analysis of selected gene and enhancer lines, fish were out-crossed and their resulting embryos were placed in individual wells, following which DNA was extracted and sequenced. For unselected fish, DNA from groups of six injected fish was placed in one well and sequenced.

NGS of selected Ds lines produced 5473 putative inserts with fragment counts ranging from 5–10,000. To obtain a high-confidence integration set, we performed PCR validation of a subset of NGS-identified sites. We also examined the sequencing results of single insert lines and obtained NGS fragment counts for TAIL-identified sites. Based on these observations, a putative integration site was deemed high-confidence if it was detected with > 50 counts.

Based on findings with the selected set, we devised similar criteria for the unselected Ds set, although we lowered the general fragment count cut-off to 7 as we expected inserts to be diluted in each sample. We noticed that recognition sequences for restriction enzymes used during the NGS protocol were highly prevalent in single fragment putative inserts. Therefore, we applied a more stringent cut-off criterion of 50 fragment counts for these sites, so that no more than 10% of the total sites contained the restriction enzyme recognition sequences.

### Tol2 and MMLV integration sites and matched controls

Inverse-PCR and TAIL-PCR results for Tol2 integrations were obtained from published gene and enhancer trap screens (Kawakami *et al.* 2010; Kondrychyn *et al.* 2011; http://kawakami.lab.nig.ac.jp/ztrap/; http://plover.imcb.a-star.edu.sg/), and mapped to the Zv9 genome assembly as described above. MMLV retrovirus integration sites generated by NGS were obtained from the Zebrafish insertion collection (Varshney *et al.* 2013; http://research.nhgri.nih.gov/ZInC/). We used various matched control sets for comparison, taking into account the sequencing technique, genome mapping, and size of the different experimental integration sets. In the case of Tol2 and Ds integration sites obtained by inverse-PCR and TAIL-PCR, we generated one million 50 bp random genomic locations using the BEDTools random tool (Quinlan and Hall 2010), and mapped them back into the Zv9 genome assembly using Bowtie (Langmead *et al.* 2009) to remove regions mapping to multiple locations. We then performed 1000 independent random samplings of these regions to produce control sets of the same size as the experimental sets. For NGS-generated insertions, we replicated the conditions of the sequencing protocol and took account of repetitive regions of the genome (LaFave *et al.* 2014). Briefly, we identified the location of all *Mse*I, *Bfa*I, and *Csp*6I restriction enzyme sites across the genome. We then calculated the distance from each integration to the nearest of the three restriction sites that could have produced a mapable fragment. We used these distances to generate files containing one matched random integration of the same distance and same restriction site as each experimental integration. The corresponding sequences were then aligned back to the Zv9 assembly with Bowtie using the same settings as in the experimental workflow, repeating this process 1000 times. In this way, the random sites take into account two potential sources of bias: distance from restriction sites and alignability of the read. The selected and unselected Ds, Tol2, and MMLV integration sites used in our analysis are presented in Supporting Information, Table S1.

### Bioinformatic analyses

Integration sites and control sets were compared with the various genomic features using BEDTools intersect (Quinlan and Hall 2010). Genomic features were considered overlapping if they shared at least 1 bp of the insertion site. Location of CpG islands and repetitive elements were obtained from the UCSC browser track. H3K4me1 and H3K4me3 hotspots were obtained from Aday *et al.* (2011), and CpG DNA methylation from McGaughey *et al.* (2014). Gene models were obtained from the Ensembl database. Where appropriate, standard nomenclature was followed (Mullins 1995). Gene ontology analysis was performed with DAVID (Huang *et al.* 2009a,b).

Ensembl gene annotations were used for determining integration site distribution across gene regions, with different features obtained from the UCSC track. Integrations were assigned to a gene when they occurred anywhere between 5 kb beyond the transcription start site (TSS) and the transcription termination site (TTS). All genes were counted when multiple genes overlapped the integration sites. Similarly, all features were counted when multiple gene features overlapped the integration sites. Intergenic regions were defined as lying beyond 5 kb from TSS or TTS. To look for distribution along a gene region, gene size was normalized to 100%. To study the distribution of insertion sites across the TSS and TTS, the distance from site was obtained within a 1 kb window around the gene feature.

Gene expression information was obtained from previously published RNA-seq experiments (Harvey *et al.* 2013). To estimate overall gene expression levels, we combined the expression level in FPKM (fragments per kb of transcript per million mapped reads) for each gene across developmental time-points. Sites were assigned to genes within ± 5 kb of the TSS and TTS.

### Statistical analyses

We carried out genomic feature preference analyses by bootstrapping, searching for values of a given random control data set that differed from the corresponding value in the experimental set. For a given genomic feature, the enrichment value is the ratio of its prevalence in the experimental set over each of the matched control sets averaged over the total (n = 1000). To calculate the *P*-value, we counted the number of random sets in which a particular feature was enriched or depleted in relation to the experimental set, and divided the total by 1000 (the number of random tests). *P*-values were calculated for both enrichment and depletion in every category, although we only report the relevant *P*-values here. The significance threshold is *P* = 0.05.

### Mouse cell integrations

Retrovirus and transposon integrations in mouse cells were obtained from published datasets (de Jong *et al.* 2014). Specifically, 131,594 Sleeping Beauty (SB) integrations and 1,222,667 piggyBac (PB) integrations into mouse embryonic stem cells (mESC), together with 180,469 mouse mammary tumor virus (MMTV) integrations into mouse mammary gland cells (NMuMG), were analyzed for sequence and structural motif discovery, using random integration sites as controls.

### Motif and structural feature discovery

We obtained 48 bases of flanking sequences from the zebrafish Zv9 or mouse MM10 genome assemblies for motif analyses, preserving the orientation of insertion. In the case of Ds and Tol2 integrations we obtained 20 bases before and after the 8 bp duplicated site. For zebrafish MMLV or mouse SB, PB, and MMTV sites, we obtained 20 bases before and 28 bases after the insertion site, so that position 21 was always the first position of the integration site. Consensus motifs were generated with weblogo v3 and displayed as bits or probability (Crooks *et al.* 2004).

We used crystallography-derived values to calculate six DNA strand movements (Rise, Roll, Shift, Slide, Tilt, and Twist) around the integration sites. Using a custom Python script, we reduced each 48 bp sequence to its underlying dinucleotides, such that position 1 and 2 represented the first dinucleotide, position 2 and 3 represented the second dinucleotide and so on. Each dinucleotide was then assigned the corresponding movement values deduced from crystallography data (Olson *et al.* 1998). For example, a GC dinucleotide would produce an average of 36.1 degrees of Twist, and 0.41Å of Slide, while a GG dinucleotide would correspond to 32.9 degrees of Twist and –0.22Å of Slide. These movement values were then averaged for each dinucleotide position and plotted.

### Data availability

The authors state that all data necessary for confirming the conclusions presented in the article are represented fully within the article.

## Results

### Generation of high-confidence Ds integration sites

**Figure 1:**
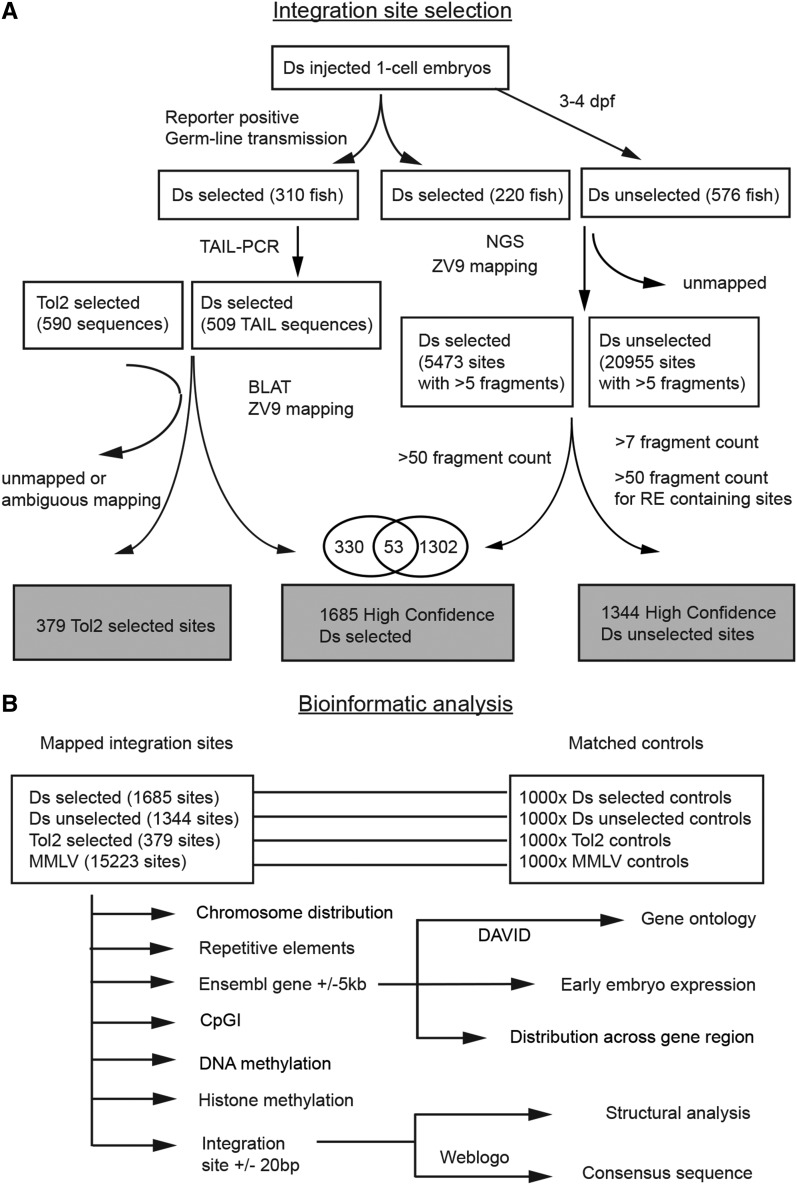
Experimental design. (A) Pipeline for obtaining high-confidence selected and unselected Ds integration sets. Ds integrations were generated by injection of Ds trapping plasmid together with Ac transposase capped-RNA at 1-cell stage. Following injection, larvae were collected at 3–4 days post fertilization (dpf) without selection to obtain a set of unbiased Ds integrations, or raised to adulthood, outcrossed, and selected for reporter gene expression. Ds integrations were analyzed by thermal asymmetric interlaced PCR (TAIL-PCR) and genomic sequencing. Numbers in parenthesis (*i.e.*, 310, 220 and 576) represent the number of fish lines or injected embryos from which the Ds integration sites were identified. (B) Data analysis scheme. Selected and unselected Ds integrations were compared with Tol2 and MMLV (Moloney Murine Leukemia Virus) sites. One thousand controls were generated for each integration dataset. RE, restriction endonuclease; Zv9, zebrafish reference genome.

Based upon the experimental strategy shown in Figure 1, we generated two sets of Ds integration data for our genome-wide analysis, one “selected” and one “unselected.” For the source of “selected” Ds sites, we analyzed zebrafish lines obtained as part of the FISHTRAP mutagenesis screen (http://fishtrap.warwick.ac.uk, Quach *et al.* 2015). These stable transgenic lines were selected on the basis of expression of fluorescent reporters (mCherry and/or GFP) during the first 7 d of development and represent protein/enhancer trap events. To identify the Ds insertion sites, we first performed thermal asymmetric interlaced PCR (TAIL-PCR) on 310 reporter positive fish lines generating 385 unique insertion sites. TAIL-PCR results represented only ∼75% of sites estimated by Southern blot analysis (Figure S1), so we performed NGS on 106 of these lines and 114 additional lines, obtaining a total of 1685 unique high-confidence Ds integration sites from 424 zebrafish lines.

Remarkably, only ∼45% of the TAIL-identified flanking sites were also identified by NGS. Both TAIL-PCR and NGS rely on unambiguous mappings to the genome assembly for integration site identification. However, we found that TAIL-PCR produced longer flanking site sequences and was more accommodating of mismatches between the Tubingen-strain reference genome and the AB strain used in the Ac/Ds mutagenesis screen (Howe *et al.* 2013; Quach *et al.* 2015). In fact, 133 out of the 385 inserts identified by TAIL-PCR differed substantially from the genome assembly and were not mapped by NGS, explaining the limited overlap between the two techniques. In addition, some previously identified TAIL-PCR sites were detected at very low levels in the NGS results (Figure S2), suggesting that different flanking sequences might be more efficiently identified by the two techniques.

Since each selected fish line contains on average 3–4 insertions, some of which are likely unrelated to the fluorescent reporter expression pattern used to identify the line, we expect the selection bias to be mitigated in our dataset. However, these selected Ds inserts would still have to be incorporated into the germ-line for stable transmission. Therefore, in order to obtain an unbiased set of Ds integration sites, we performed NGS on 576 embryos that had been injected with the Ds plasmid and Ac transposase mRNA, but not selected on the basis of transgene reporter expression. In total, we obtained a set of 1344 high-confidence integrations which we used as our “unselected” Ds set. These two Ds insertion sets were compared with 15,223 unselected MMLV retroviral insertions (Varshney *et al.* 2013) (Table 1), and analyzed together with appropriately matched control sets of the same size and mapping characteristics. Although limited by the smaller sample size and various selection strategies, we also examined the distribution of 379 Tol2 integrations combined from two published enhancer and protein trap datasets (which we henceforth refer to as the “selected Tol2” set) (Kawakami *et al.* 2010; Kondrychyn *et al.* 2011).

**Table 1:** Integration datasets analyzed in this work

Database	Model	Selection	Germ-Line Integration	Stage Injected	Detection Technique	Mapped Sites	Reference
Ds selected	Zebrafish	Yes	Yes	1-cell	TAIL-PCR	383	(Quach *et al.* 2015)
					NGS	1355	This work
					Total	1685	
Ds unselected	Zebrafish	No	No	1-cell	NGS	1344	This work
Tol2 selected	Zebrafish	Yes	Yes	1–2 cell	Inverse-PCR	75	(Kawakami *et al.* 2010)
		Yes	Yes	1–2 cell	TAIL-PCR	304	(Kondrychyn *et al.* 2011)
					Total	379	
MMLV	Zebrafish	No	Yes	1000–2000 cell	NGS	15,223	(Varshney *et al.* 2013)
Sleeping beauty	mESC	No	N/A	N/A	NGS	131,594	(de Jong *et al.* 2014)
PiggyBac	mESC	No	N/A	N/A	NGS	122,667	(de Jong *et al.* 2014)
MMTV	Mouse mammary cells	No	N/A	N/A	NGS	180,469	(de Jong *et al.* 2014)

TAIL-PCR, thermal asymmetric interlaced PCR; NGS, next-generation sequencing; MMLV, Moloney Murine Leukemia Virus; mESC, mouse embryonic stem cells; MMTV, Mouse Mammary Tumor Virus.

### Ds integration sites are distributed throughout the zebrafish genome

**Figure 2:**
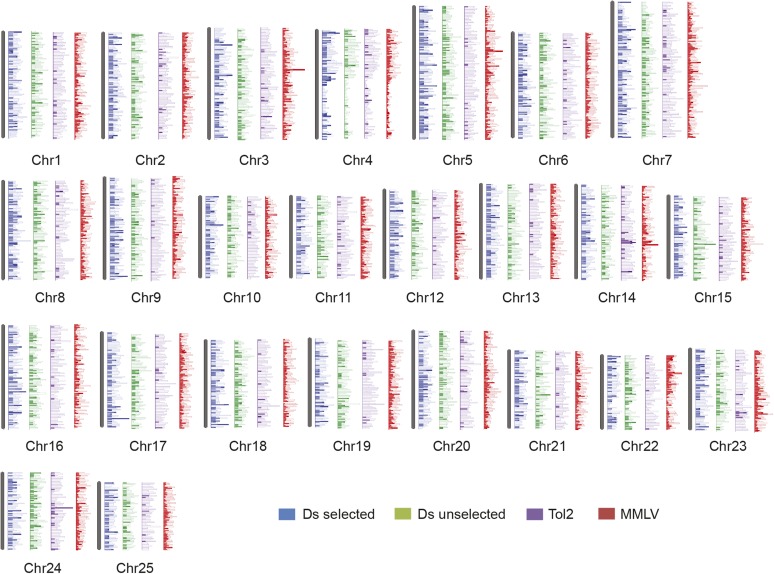
Insert distribution across the zebrafish genome. Ds, Tol2, and MMLV (Moloney Murine Leukemia Virus) integration sites were distributed across all chromosomes, with regions of relative over and underrepresentation. Solid bars represent integration sites. Open bars represent 20,000 matched control sites.

To investigate integration site preferences, we first examined their distribution across the zebrafish genome at the chromosome level (Figure 2). MMLV, selected Tol2, and selected and unselected Ds integrations were found distributed across all 25 chromosomes. Consistent with some of the Tol2 integration sites having been created by remobilization of existing genomic integration sites, we observed enrichment of Tol2 sites close to donor locations in chromosomes 14 and 24 (Kondrychyn *et al.* 2011). Although some chromosomal regions appeared to be either over or underrepresented when compared to matched controls, in general, Ds, Tol2, and MMLV integrations were all found widely distributed across the genome, and we did not observe integration rich regions shared across any of the datasets.

**Figure 3:**
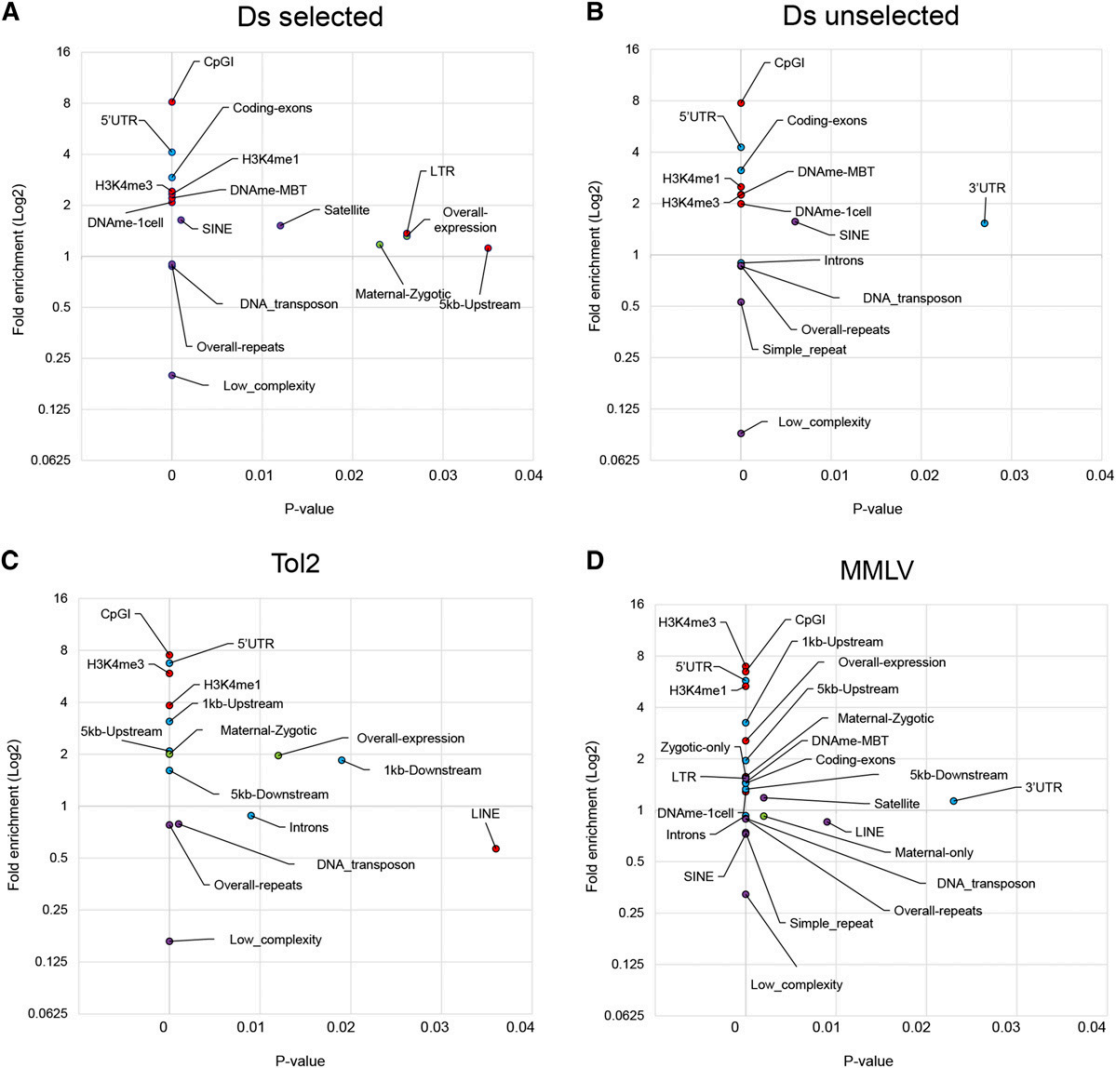
Summary of genomic features analyzed. Genomic features analyzed for (A) Ds selected, (B) Ds unselected, (C) Tol2, and (D) MMLV (Moloney Murine Leukemia Virus). Average fold enrichment values (representing 1000 ratios of experimental over match controls) plotted on the y-axis. *P*-values plotted on the x-axis. Statistically significant enriched/depleted features with *P*-value < 0.05 are presented (detailed results are provided in Table S3).

To explore if specific features of the genome were correlated with integration events we analyzed the overlap of 25 genomic features with the integration datasets (Table S2). A plot of the statistically significant enriched and depleted genomic features is presented in Figure 3 (with the full results shown in Table S3). In the following sections we discuss the main findings.

### Ds insertions show preference toward gene regions

**Figure 4:**
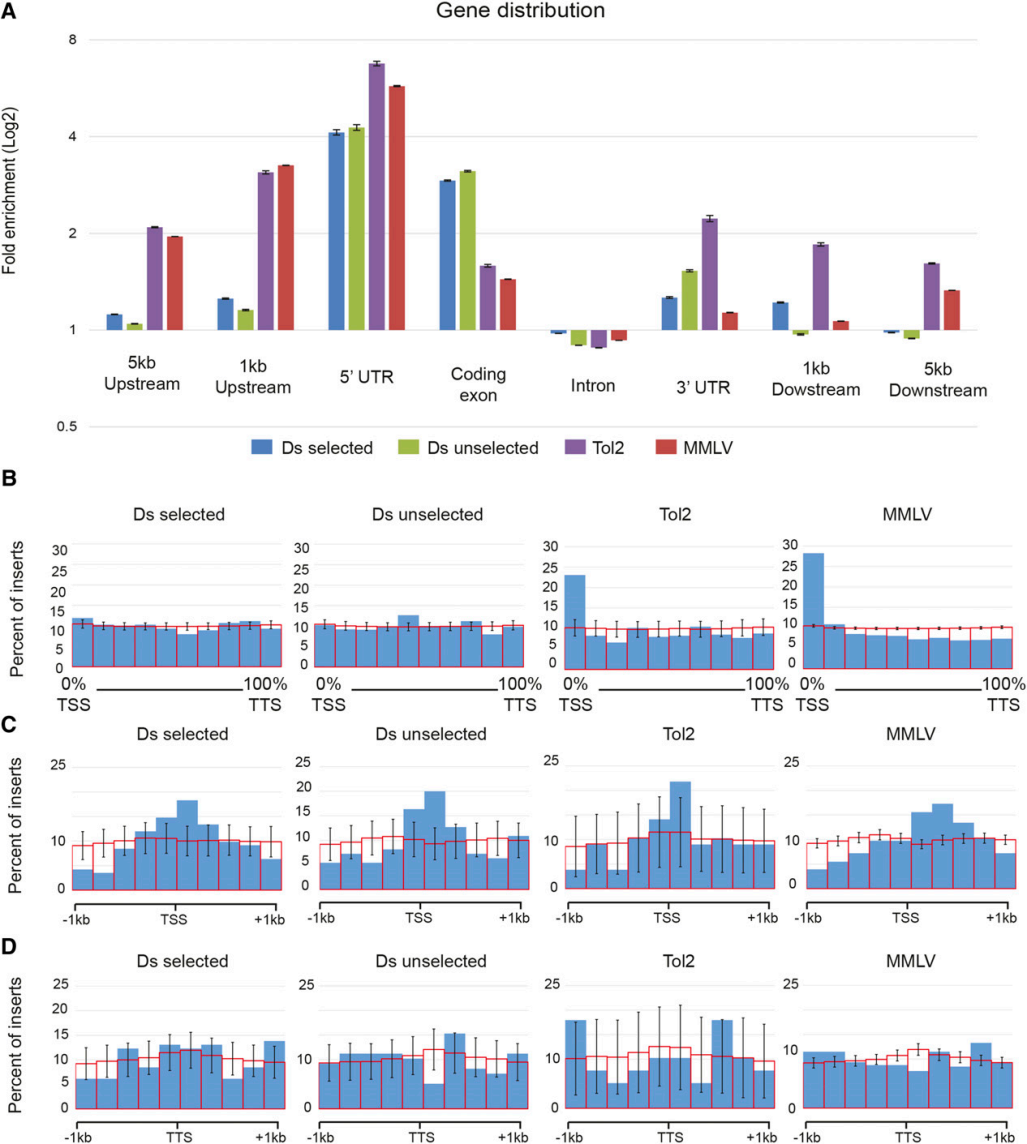
Distribution of integration sites across gene regions. (A) Fold enrichment values for various gene subregions. Average enrichment ± standard error (n = 1000). Ds integrations show preference for coding and 5′ gene regions. Tol2 and MMLV (Moloney Murine Leukemia Virus) integrations show preference for 5′ regions of genes. (B) Ds integrations are distributed uniformly across the length of gene regions, while Tol2 and MMLV are enriched toward the first 10% of genes. (C) Ds, Tol2, and MMLV show enrichment close to transcription start site (TSS), but are not enriched around the transcription termination site (TTS) (D). Solid blue bars represent integration sites. Open red bars represent average of 1000 matched controls ± standard deviation.

We first examined whether Ds, Tol2, and MMLV integrated preferentially in gene regions. We obtained Ensembl gene prediction coordinates and identified gene features such as introns, exons, and UTRs. Any integration occurring beyond 5 kb of the transcriptional start and termination sites was deemed intergenic. We found that Ds, Tol2, and MMLV integrations were enriched around gene regions, but with differential preference for specific gene features (Figure 4A). Specifically, Ds integrations showed a preference for coding exons, whereas Tol2 and MMLV preferentially integrated in the 5′-UTR and regions up-stream of the TSS. In general, we observed no differences in the preference of selected and unselected Ds integrations for intronic, 5′-UTR, 3′-UTR, and intergenic regions. Detailed examination of sites that overlapped gene regions showed that while MMLV and Tol2 integrations are biased toward the first 10% of the gene area, Ds integration sites are more broadly distributed throughout gene regions (Figure 4B).

Because of the preference of Tol2 and MMLV for the 5′ region of genes, we examined the distribution of integrations around the TSS (Figure 4C). MMLV integrations were significantly biased downstream of the TSS, consistent with previous observations in human cells that identified MMLV integrations going into enhancers within the 1^st^ intron (Wu *et al.* 2003; LaFave *et al.* 2014). In contrast, both Tol2 and Ds integrations show a symmetrical distribution around the TSS, although the number of transposon integrations was smaller than those for MMLV. We found no similarity in integration patterns around the TTS for Ds, Tol2, or MMLV. Taken together, we found that Ds, Tol2, and MMLV integrations have a preference for gene regions. Ds integrations were found more broadly distributed along genes, unlike Tol2 and MMLV, which showed a significant bias toward the 5′ region of genes.

### Integrations are correlated with measures of gene and enhancer activity

Next, we asked whether the genes targeted by transposon and retroviral integrations shared any common characteristics. For this analysis, integrations were assigned to a particular gene if they occurred within 5 kb of their TSS and TTS. Although a few genes were targeted by more than one type of integration, these tended to span large regions of the genome. Gene ontology (GO) analysis on genes targeted by Ds integrations did not reveal any significant enriched categories (Table S4 and Table S5). In contrast, a number of Tol2 integrations targeted Hox genes (Table S6), specifically hoxa5a, hoxa3a, hoxd3a, hoxc5a, hoxd4a, and hoxc3a (GO analysis, Benjamini *P*-value = 0.015). Since these genes are in chromosomes 9, 19, and 23, this preference cannot be accounted for by local hopping from donor sites on chromosomes 14 and 24. Some genes were targeted two or more times by MMLV integrations. These genes were significantly enriched (Benjamini *P*-value < 0.001) for GO categories representing biological processes occurring during gastrulation, such as cell migration, regulation of transcription, and embryonic morphogenesis (Table S7).

To test if integration was correlated with gene activity, we calculated the gene expression levels of the genes targeted by integrations (Figure 3 and Figure S3). We measured the median expression level of genes across various time-points as well as an overall expression level (the sum of the individual expression) using published RNA-seq data (Harvey *et al.* 2013). We noticed that, on average, genes targeted by Tol2 and MMLV tended to have higher expression in the early embryo (Figure S3). In contrast, genes targeted by Ds did not show any gene expression difference from matched controls.

We also analyzed whether these genes shared any particular pattern of expression, categorizing the genes as having maternal only expression, zygotic only expression, or both maternal and zygotic expression (maternal-zygotic) (Figure S3). Ds integrated similarly in genes with each of these expression patterns. In contrast, Tol2 and MMLV integrated preferentially into zygotically expressed genes, and not maternal genes. Since MMLV injections were performed at the 1000 to 2000 cell stage and after zygotic genome activation, our GO and gene expression analyses are consistent with MMLV preferentially integrating into actively transcribed genes from the onset of zygotic genome activation.

Epigenetic marks involved in gene regulation, such as histone modification and differential DNA methylation, might facilitate integration into specific gene regions (Potok *et al.* 2013). Although the overall frequency of integrations within CpG islands was low, we found an increased preference of all integrations toward CpG islands both within and outside promoters, suggesting that regulated regions are preferred regardless of the integration vector used (Figure S4). Similarly, while DNA methylation status was not a key determinant of integration preference, regions of higher CpG DNA methylation at 1-cell and midblastula transition (MBT) stages of development showed a twofold increase in Ds integrations compared to matched controls (Figure S4). This correlation was found for DNA methylation both within and outside gene regions.

Next, we examined whether integrations overlap with chromatin modifications marking active promoter and enhancer elements (Figure S4). All insertion sites, including those that do not overlap with gene regions, preferentially targeted regions rich in chromatin modifications associated with active promoters and enhancers (tri and monomethylation of Histone 3 Lysine 4) suggesting that integration sites in intergenic regions might represent unannotated genes or novel enhancer elements.

### Integrations in repetitive sequences

Repetitive elements, which account for 52.2% of the zebrafish genome (Howe *et al.* 2013), have been shown to have roles in chromosome structural organization, gene regulation, genome integrity, and evolution (Kidwell and Lisch 2000; Lander *et al.* 2001; Waterston *et al.* 2002; Feschotte 2008; Ting *et al.* 2011; Zhu *et al.* 2011). Therefore, we examined the integration preferences for the various repetitive element families, such as DNA transposons, LINEs, and SINEs, present in the zebrafish genome (Figure S5). DNA transposon sequences and low-complexity repeats were consistently underrepresented in sites of integration. However, we observed varying preferences for the other types of repetitive elements. Specifically, we noticed that LTR sequences were overrepresented in MMLV integration sites, while SINE, LINE, and simple repeats were underrepresented. In contrast, we observed a weak overrepresentation of Ds integration sites overlapping SINE and LTR elements, but no significant over or underrepresentation of sites overlapping simple repeats.

### Ds and Tol2 target sites do not show a strict sequence motif

**Figure 5:**
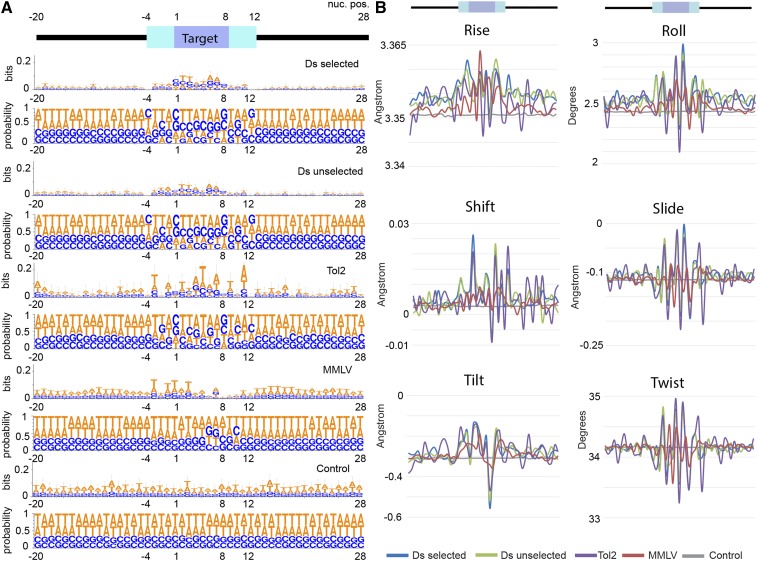
Target sites show structural features even in the absence of a strict motif. (A) Graphical representations of nucleic acid multiple sequence alignment were generated with WebLogo v3.4 (Crooks *et al.* 2004). Ds and Tol2 integrations show weak preference for specific nucleotides at the integration site. Numbers on top indicate nucleotide position around the integration site shown on the x-axis. Information measured in bits and probability is shown on the y-axis. (B) Average values of protein-DNA movement for each position in the multiple sequence alignment plotted according to their values. Numbers on top indicate position around the integration site shown on the x-axis. Red lines represent integration site data. Blue lines denote the average of matched controls (n = 1000). MMLV, Moloney Murine Leukemia Virus.

We then examined whether any features at the sequence level could help predict Ds, Tol2, and MMLV integrations in the zebrafish genome. In contrast to the strong binding site preference for the element ends, no strong target site consensus sequences have been identified for Ds and Tol2. However, a weak preference for specific nucleotides at the target site has been reported. Previous analysis of Ds integrations in maize suggested the presence of a weak palindromic consensus sequence at the target site (Vollbrecht *et al.* 2010), while Tol2 integrations in zebrafish suggested the presence of a TNA(C/G)TTATAA(G/C)TNA motif (Kondrychyn *et al.* 2009). Therefore, we searched for a consensus sequence at the target site in our Ds data and in the MMLV dataset (Figure 5A).

**Figure 6:**
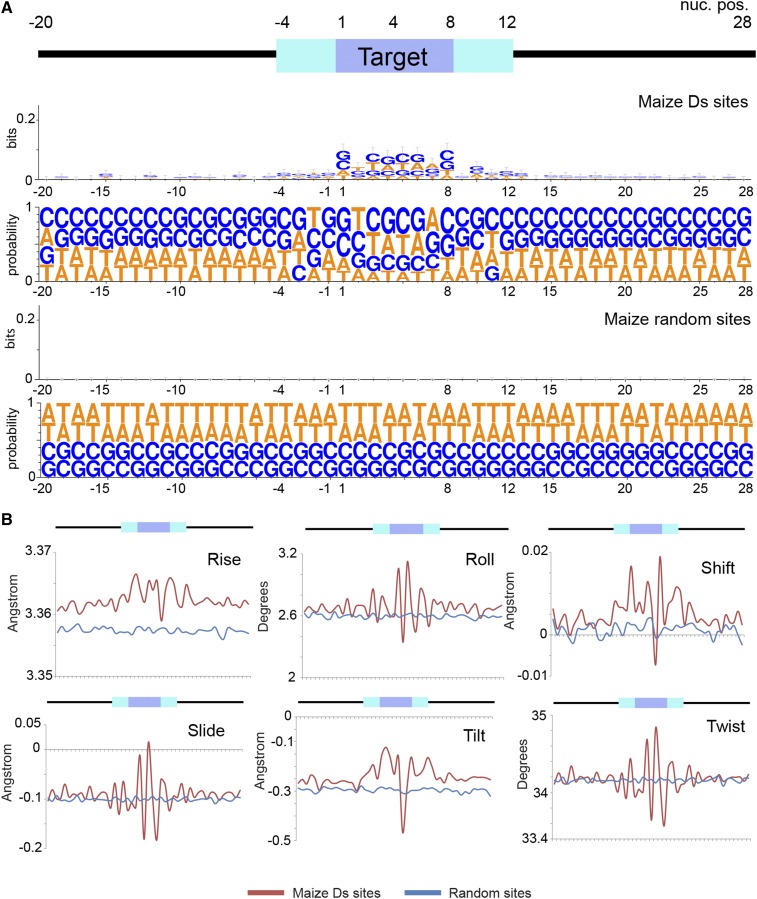
Ds integration site analysis in maize. 1826 Ds integration sites were obtained from published datasets (Vollbrecht *et al.* 2010) and compared against random integration sites. (A) Sequence logo for Ds integrations. (B) Structural features of DNA at integration sites.

Consistent with previous reports, MMLV integrates preferentially in AT-rich regions (Wu *et al.* 2005; LaFave *et al.* 2014), and we also observed a region of relative AT depletion following the integration site. In our analyses, we were able to detect the weak consensus sequence previously reported for Tol2 (Kondrychyn *et al.* 2009). We did not observe the weak sequence motif for Ds integration sites previously observed in maize (Figure 6). However, when insertion site sequences were aggregated, we noticed a weak palindromic motif spanning 14 bases around the Ds insertion site with consensus similar to Tol2. This sequence is seldom found within the dataset target sequences *per se*, and appears only when insertion sites are aggregated.

### Transposon and retroviral integration sites show similar structural features

Local interactions between adjacent nucleotides can induce distortions in the regular double helix structure (Olson *et al.* 1998). To test whether integration sites were more likely to be deformed by protein-DNA interactions, we used data from protein-DNA complexes to calculate six structural features of DNA: Rise, Roll, Shift, Slide, Tilt, and Twist. For example, protein DNA-twist predicts the twist angle torsion between adjacent bases, so that a dinucleotide pair with a high value of protein-DNA twist is more likely to be deformed by protein–DNA interaction than one with a lower value. For transposon integrations, we observed significant changes from normal for these six features arranged in a symmetrical pattern around the target sites (Figure 5B). For retroviral integrations, the outlying values extended a few bases downstream of the target site. Regardless of the vector used, all integrations appear to fall in regions of higher DNA flexibility.

**Figure 7:**
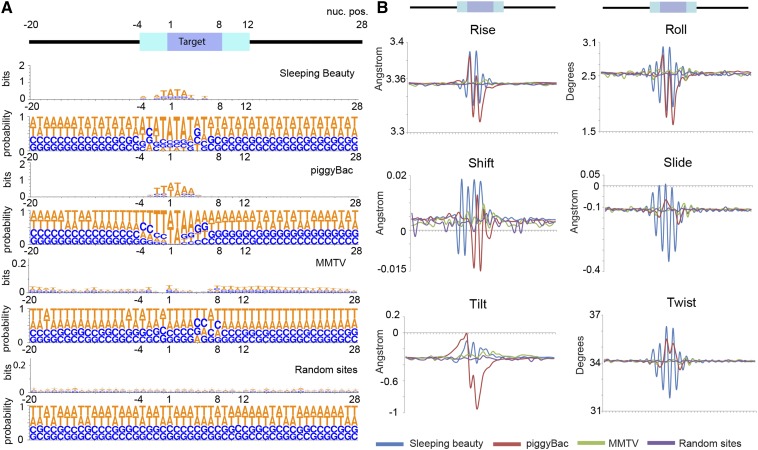
Measures of DNA flexibility show similar features in transposon and retroviral integration sites in mouse cells. (A) Sequence logo for various integrations in mouse cells show an obvious common motif. Numbers on top indicate position around the integration site shown on the x-axis. Information measured in bits and probability shown on y-axis. (B) Structural features of DNA at integration sites. Average DNA flexibility values shown in y-axes. Nucleotide position shown on x-axis. MMTV, Mouse Mammary Tumor Virus.

To determine if this feature is specific to integrations in zebrafish or whether it is found in other vertebrate genomes, we analyzed previously reported integration sites for Sleeping beauty and piggyBac transposons in mouse ESCs, and mouse mammary tumor retrovirus (MMTV) in mouse mammary cells (de Jong *et al.* 2014) (Figure 7A). Sleeping beauty was found to target TATA sequences and piggyBac insertions fell in AT-rich regions. In contrast, no consensus sequence motif was observed for MMTV retroviral insertions. Nonetheless, all integrations occurred at regions of high DNA flexibility (Figure 7B). Taken together, our findings suggest that structural features in the target DNA are common in transposon and retroviral integration sites in vertebrate genomes, and can potentially be used to enhance the efficiency of genome engineering by these and other methods.

## Discussion

Transgenesis is a powerful tool which, coupled to new genome editing techniques, continues to make zebrafish an excellent model organism in which to perform functional genomic studies. In this study, we set out to compare the integration preferences of three popular tools for generating transgenics (MMLV retrovirus, and Ds and Tol2 transposons). We used a combination of TAIL-PCR and NGS to detect Ds integration sites. Only ∼45% of Ds sites identified by TAIL-PCR were captured by NGS. Many of the Ds integration sites that were not detected by genomic sequencing showed variation from the genome assembly sequence, suggesting they were not mapped under the parameters used. Ds sites showed a wide range of detection efficiency as measured by average fragment counts produced. Differences in the distance of the insertion site to the restriction enzyme cut site used during the NGS protocol, or the efficiency of PCR amplification, could potentially explain the different isolation efficiencies observed.

The use of NGS for mapping made it possible to identify integrations in the absence of germ-line transmission or reporter expression. We found selected and unselected Ds sites to have remarkably similar integration characteristics. Because each selected fish line contains ∼3–4 insertions, it is possible that some of the selection bias could have been mitigated in our Ds “selected” dataset. However, we did not observe a significant difference in genomic feature overlap between selected lines harboring one *vs.* multiple insertions either. Therefore, the similarity between selected and unselected insertions is unlikely to be explained by the number of insertions per line alone (Figure S6).

Despite the small sample size of Tol2 integrations and different selection strategies in the various screens, we found that all vectors showed a preference for gene regions. Tol2 and MMLV were largely concentrated around the 5′ regions of highly expressed genes. In contrast, we found that Ds sites were enriched in coding regions, and broadly distributed along gene regions, matching regions of high DNA methylation outside of promoters. Significantly, Ds integration preference for gene regions was found even in the absence of selection.

The zebrafish genome shows an overall repeat content of 52.2%, the highest reported so far in a vertebrate (Howe *et al.* 2013). Consistent with this, integrations frequently overlapped repetitive elements. Analysis of repetitive elements in the zebrafish genome revealed that LTRs, low complexity, and simple repeats are more likely found in coding gene regions, while SINE elements are more likely present in 5′-UTRs (Figure S7). Our matched control sets have similar mapping characteristics to the experimental integration sets, therefore differences in mapping cannot account for the relative depletion of low complexity repeats within integration sites. The preferential integration into specific repetitive elements could represent a preference for their specific underlying sequences or the resulting structural characteristics. Alternatively, other genomic characteristics could be correlated with the different repetitive elements. Repetitive sequences show differential methylation and activity in zebrafish (McGaughey *et al.* 2014). DNA repeats have also been shown to be transcribed, and have been suggested to provide regulatory elements to protein-coding genes (Wang *et al.* 2007b; Bourque *et al.* 2008; Faulkner *et al.* 2009; Tyekucheva *et al.* 2011). Moreover, binding sites for important regulatory factors such as CTCF or TP53 are often associated with genomic repeats (Wang *et al.* 2007b; Bourque *et al.* 2008; Chadwick 2008; Simeonova *et al.* 2012).

An important question concerns the presence of particular insertion hotspots, since integration can cause adverse events such as activation of proto-oncogenes or inactivation of essential cellular genes. Both Tol2 and MMLV showed enrichment for specific GO categories. Ds integrations showed no observable correlation with specific gene types. While no single transgenic tool will be equally suited for every experimental inquiry, our analyses should help in the choice of transgenic system for interrogating gene function.

At the sequence level, both Ds and Tol2 target sites shared a similar weak motif that appeared only when sequences were aggregated. The weak motif likely reflects structural features of the target DNA. Current genome editing methods rely exclusively upon nucleotide sequence for selection of targeting sites (Lim *et al.* 2013; Irion *et al.* 2014). Our analysis of known transposon and retroviral integration sites in mouse ES cells and in zebrafish shows that regions of higher DNA flexibility are preferred for integrations of exogenous sequences. Thus, structural features in DNA influence the site of insertion in vertebrate genomes. This feature can potentially be used in combination with sequence information to enhance the efficiency of genome editing, and to improve precision engineering at desired locations within genomes.

We found the presence of DNA flexibility features to be conserved among different types of integrations and in different species. However, the specific features differ from system to system, likely resulting from differences in their mechanisms of integration, or the presence of different cofactors. The ability of hAT transposons to function in diverse species suggests that they might not require specific cofactors, or rely on very highly conserved cofactors (Weil and Kunze 2000; Emelyanov *et al.* 2006). In contrast, several groups have identified bromodomain and extraterminal (BET) proteins as the major host factors that specifically interact with MMLV integrase and mediate the preferential integration of MMLV near TSS (Studamire and Goff 2008; De Rijck *et al.* 2013; Gupta *et al.* 2013; Sharma *et al.* 2013).

In summary, our genome-wide analysis shows that Ds integration sites in the presence or absence of selection are remarkably similar and can be found across the genome. A strict motif associated with target site was not found, but a preference for structural features in the target DNA was observed. Remarkably, this feature is also found in transposon and retroviral integrations in maize and mouse cells. Our findings show that structural features influence the integration of heterologous DNA in vertebrate genomes, and can facilitate efficient targeted genome engineering.

## Supplementary Material

Supporting Information
