# Supplementary material for: Genome-Wide Analysis of Transposon and Retroviral Insertions Reveals Preferential Integrations in Regions of DNA Flexibility
Source: G3 (Bethesda). 2016 Jan 26;6(4):805–17. doi: 10.1534/g3.115.026849 (PMC4825651; doi:10.1534/g3.115.026849)
Supplement: Supporting Information [file supp_6_4_805__index.html]

Genome-Wide Analysis of Transposon and Retroviral Insertions Reveals Preferential Integrations in Regions of DNA Flexibility — Supporting Information 

# Genome-Wide Analysis of Transposon and Retroviral Insertions Reveals Preferential Integrations in Regions of DNA Flexibility

## Supporting Information for Vrljicak *et al.*, 2016

**Files in this Data Supplement:**

- Figure S1 - Comparison of integration site identification by TAIL-PCR and Southern analysis. (.pdf, 362 KB)
- Figure S7 - Distribution of repetitive elements across gene regions in the zebrafish genome. (.pdf, 324 KB)
- Figure S2 - Differences in genomic sequencing efficiency for various inserts. (.pdf, 358 KB)
- Figure S3 - Expression patterns of targeted genes. (.pdf, 120 KB)
- Figure S4 - Chromatin marks are correlated with integrations. (.pdf, 152 KB)
- Figure S5 - Ds integrations show varying preferences for different repetitive elements. (.pdf, 160 KB)
- Figure S6 - Genomic feature overlap does not correlate with number of inserts per line. (.pdf, 230 KB)
- Table S1 - Integration sites analyzed (Zv9). (.xlsx, 764 KB)
- Table S2 - Genomic features analyzed. (.xlsx, 10 KB)
- Table S3 - Fold enrichment and p-values for all features analyzed. (.xlsx, 16 KB)
- Table S4 - Gene ontology analysis results for Ensembl genes hit by 1685 Ds selected integrations. (.xlsx, 37 KB)
- Table S5 - Gene ontology analysis results for Ensembl genes hit by 1344 Ds unselected integrations. (.xlsx, 26 KB)
- Table S6 - Gene ontology analysis results for Ensembl genes hit by 379 Tol2 selected integrations. (.xlsx, 17 KB)
- Table S7 - Gene ontology analysis results for Ensembl genes hit 2 or more times by 15223 MMLV integrations. (.xlsx, 92 KB)
