# Supplementary material for: Genome-Wide Analysis of Transposon and Retroviral Insertions Reveals Preferential Integrations in Regions of DNA Flexibility
Source: G3 (Bethesda). 2016 Jan 26;6(4):805–17. doi: 10.1534/g3.115.026849 (PMC4825651; doi:10.1534/g3.115.026849)
Supplement: Supporting Information [file supp_g3.115.026849_FigureS1.pdf]

Figure S1

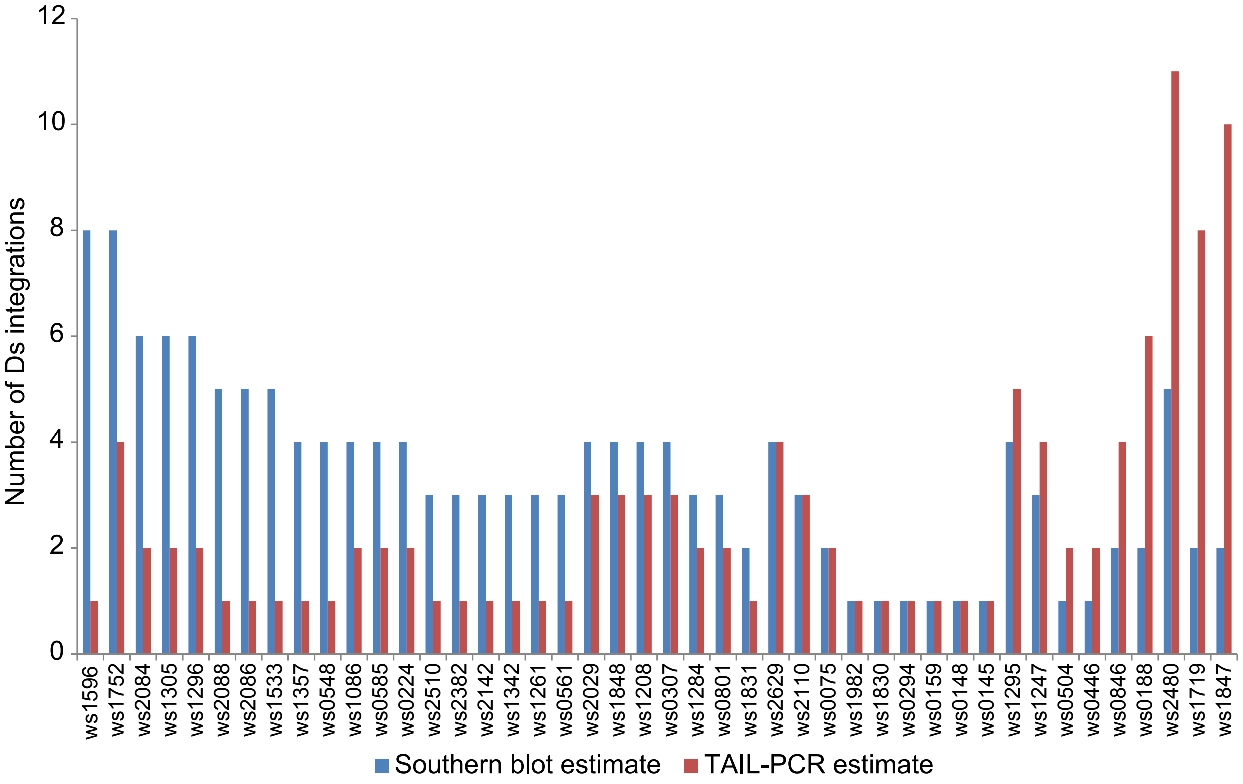

Supplementary Figure S1. Comparison of integration site identification by TAIL-PCR and Southern analysis.

Southern blot analysis was performed on a subset of selected Ds zebrafish lines. The number of Ds integrations identified by TAIL-PCR agreed with the number of inserts estimated by Southern blot analysis in only 8/44 cases. In 26/44 cases, TAIL-PCR resolved only a subset of all inserts predicted by Southern blot analysis. In 9/44 cases, more integration sites were identified by TAIL-PCR than were predicted by Southern blot analysis with a number of sites detected ambiguously in different independent lines.
