# Supplementary material for: Genome-Wide Analysis of Transposon and Retroviral Insertions Reveals Preferential Integrations in Regions of DNA Flexibility
Source: G3 (Bethesda). 2016 Jan 26;6(4):805–17. doi: 10.1534/g3.115.026849 (PMC4825651; doi:10.1534/g3.115.026849)
Supplement: Supporting Information [file supp_g3.115.026849_FigureS2.pdf]

Figure S2

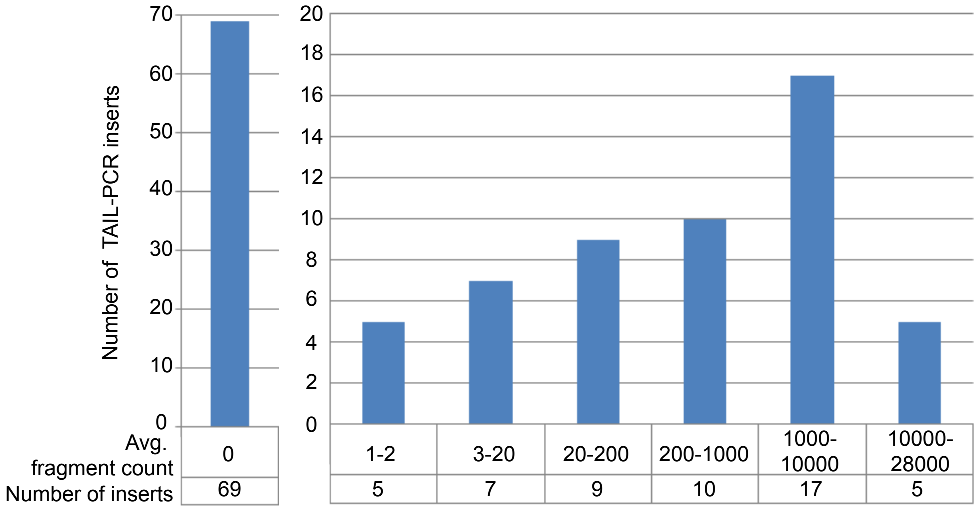

Supplementary Figure S2. Differences in genomic sequencing efficiency for various inserts. NGS fragment counts were recorded for 122 TAIL-PCR identified sites. ~45% of Ds sites identified by TAIL-PCR could be detected by sequencing. Many of the Ds integration sites that were not detected by genomic sequencing showed variation from the genome assembly sequence suggesting they could not be mapped. Ds sites showed a wide range of detection efficiency as measured by average fragment counts produced.
