# Supplementary material for: Genome-Wide Analysis of Transposon and Retroviral Insertions Reveals Preferential Integrations in Regions of DNA Flexibility
Source: G3 (Bethesda). 2016 Jan 26;6(4):805–17. doi: 10.1534/g3.115.026849 (PMC4825651; doi:10.1534/g3.115.026849)
Supplement: Supporting Information [file supp_g3.115.026849_FigureS3.pdf]

Figure S3

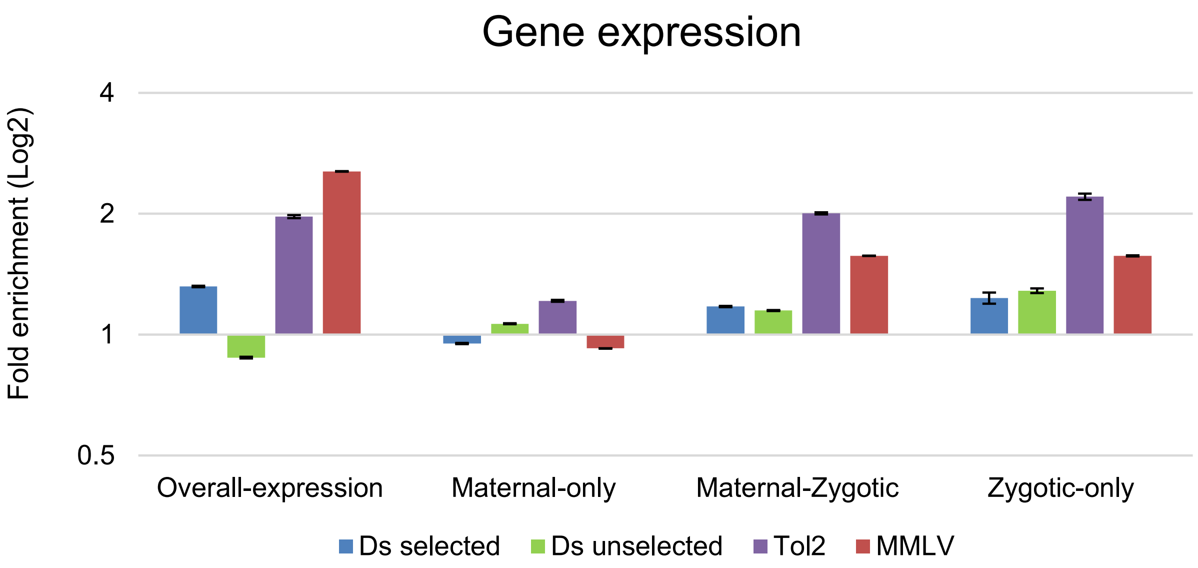

Figure S3. Expression patterns of targeted genes. Over-all expression represents median expression of genes targeted by integrations. Tol2 and MMLV preferentially targeted genes with higher expression. Gene expression pattern of the targeted genes were derived from Harvey et al. MMLV shows preference for genes with zygotic over maternal only gene expression. Fold enrichment values +/- standard error (n=1000).
