# Supplementary material for: Genome-Wide Analysis of Transposon and Retroviral Insertions Reveals Preferential Integrations in Regions of DNA Flexibility
Source: G3 (Bethesda). 2016 Jan 26;6(4):805–17. doi: 10.1534/g3.115.026849 (PMC4825651; doi:10.1534/g3.115.026849)
Supplement: Supporting Information [file supp_g3.115.026849_FigureS4.pdf]

Figure S4

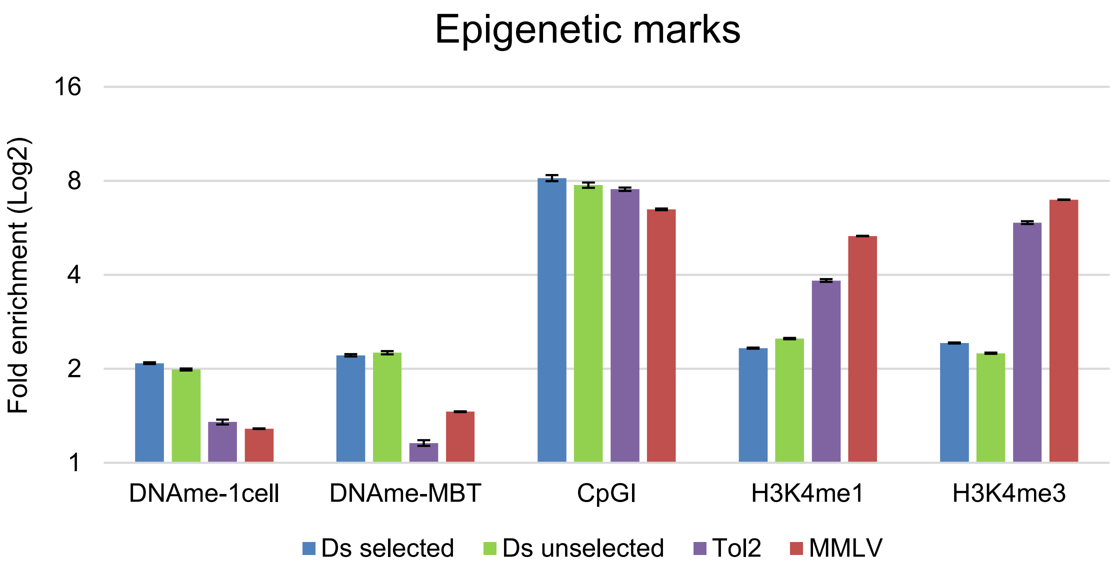

Figure S4. Chromatin marks are correlated with integrations. There is a strong preference for CpG islands, as well as CpG DNA methylation at 1-cell and MBT stages. Integration sites overlap with histone methylation marks of enhancer and promoter activity (ADAY *et al.* 2011). Fold enrichment values +/- standard error (n=1000).
