# Supplementary material for: Genome-Wide Analysis of Transposon and Retroviral Insertions Reveals Preferential Integrations in Regions of DNA Flexibility
Source: G3 (Bethesda). 2016 Jan 26;6(4):805–17. doi: 10.1534/g3.115.026849 (PMC4825651; doi:10.1534/g3.115.026849)
Supplement: Supporting Information [file supp_g3.115.026849_FigureS5.pdf]

Figure S5

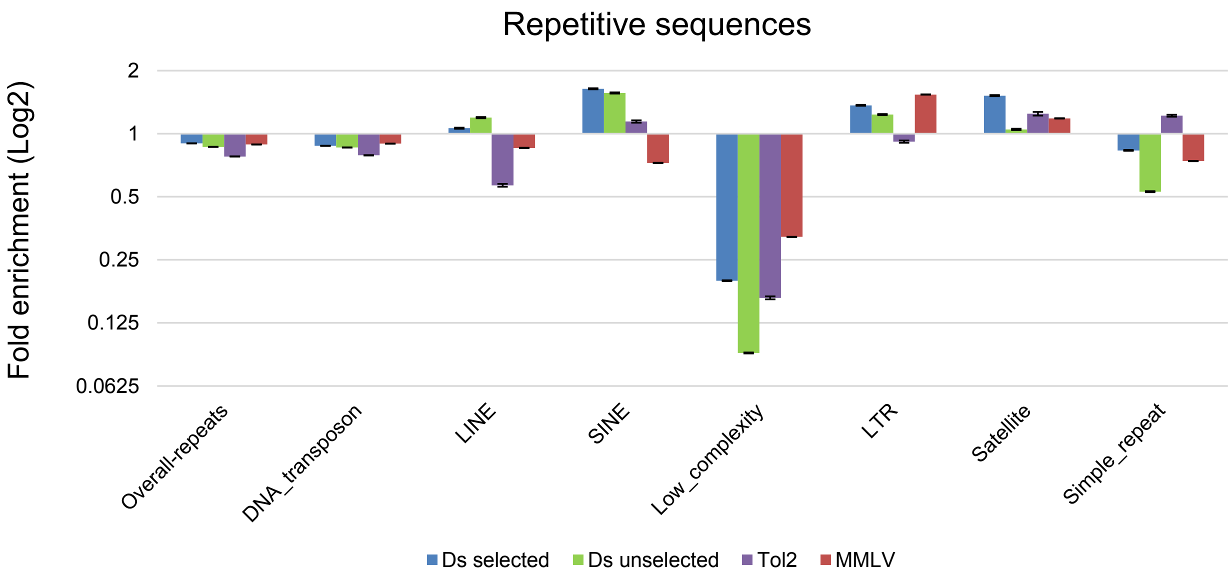

Figure S5. Ds integrations show varying preferences for different repetitive elements. Fold enrichment values for various repetitive elements. Ds, Tol2 and MMLV integrations are less likely to occur in regions with DNA transposable elements (DNA) and repeat sequences of low complexity (e.g. CT or G rich). Ds integrations show preference for LTR and SINE sequences. Fold enrichment values +/- standard error (n=1000).
