# Supplementary material for: Genome-Wide Analysis of Transposon and Retroviral Insertions Reveals Preferential Integrations in Regions of DNA Flexibility
Source: G3 (Bethesda). 2016 Jan 26;6(4):805–17. doi: 10.1534/g3.115.026849 (PMC4825651; doi:10.1534/g3.115.026849)
Supplement: Supporting Information [file supp_g3.115.026849_FigureS6.pdf]

Figure S6

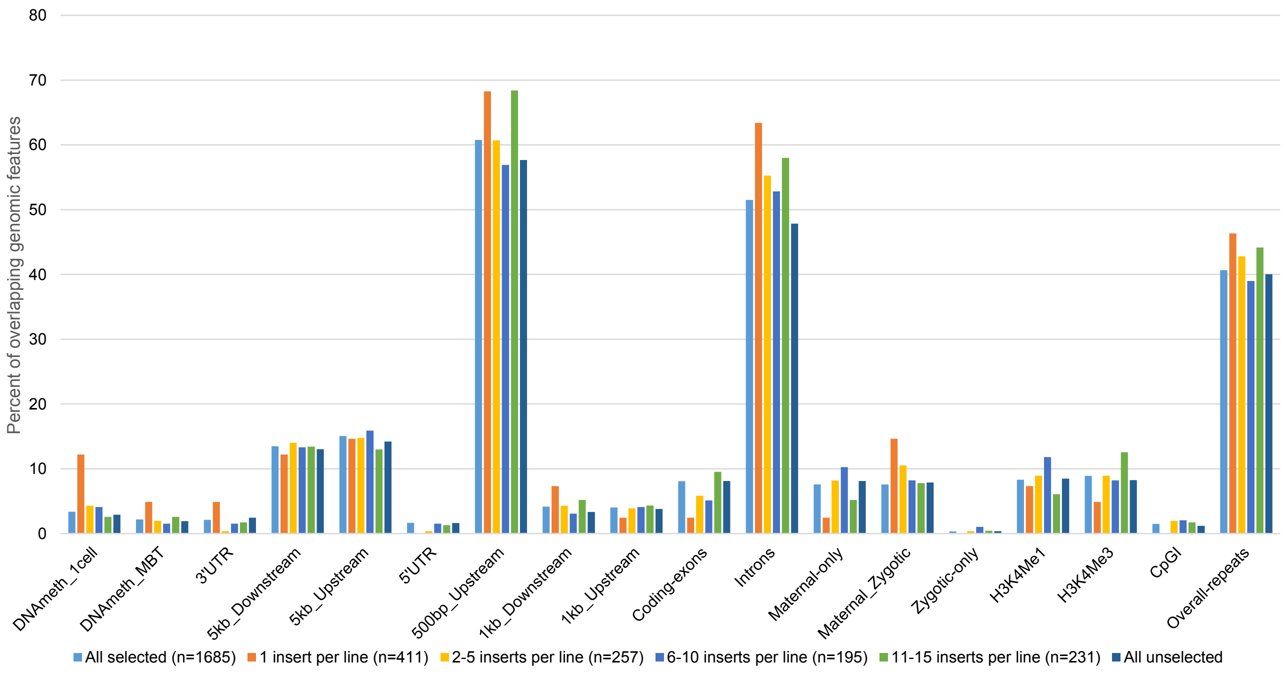

Figure S6. Genomic feature overlap does not correlate with number of inserts per line. Overlapping genomic features (%) was plotted for all Ds integrations (selected and unselected), as well as for single and multiple insert germ line Ds integration lines.
